# Dentists’ Information Needs and Opinions on Accessing Patient Information via Health Information Exchange: Survey Study

**DOI:** 10.2196/51200

**Published:** 2024-01-11

**Authors:** Shuning Li, Grace Gomez Felix Gomez, Huiping Xu, Anushri Singh Rajapuri, Brian E Dixon, Thankam Thyvalikakath

**Affiliations:** 1 Department of Dental Public Health and Dental Informatics Indiana University School of Dentistry Indianapolis, IN United States; 2 Regenstrief Institute, Inc Center for Biomedical Informatics Indianapolis, IN United States; 3 Department of Biostatistics and Health Data Sciences Indiana University School of Medicine Indianapolis, IN United States; 4 Department of Epidemiology Indiana University Richard M Fairbanks School of Public Health Indianapolis, IN United States

**Keywords:** dentistry, medical history, integrated medical and dental records, health information exchange, medical record, dental record, dental, medical information, dental care, adverse drug effect, medication, allergies, cost, data safety, data accuracy

## Abstract

**Background:**

The integration of medical and dental records is gaining significance over the past 2 decades. However, few studies have evaluated the opinions of practicing dentists on patient medical histories. Questions remain on dentists’ information needs; their perception of the reliability of patient-reported medical history; satisfaction with the available information and the methods to gather this information; and their attitudes to other options, such as a health information exchange (HIE) network, to collect patient medical history.

**Objective:**

This study aims to determine Indiana dentists’ information needs regarding patients’ medical information and their opinions about accessing it via an HIE.

**Methods:**

We administered a web-based survey to Indiana Dental Association members to assess their current medical information-retrieval approaches, the information critical for dental care, and their willingness to access or share information via an HIE. We used descriptive statistics to summarize survey results and multivariable regression to examine the associations between survey respondents’ characteristics and responses.

**Results:**

Of the 161 respondents (161/2148, 7.5% response rate), 99.5% (n=160) respondents considered patients’ medical histories essential to confirm no contraindications, including allergies or the need for antibiotic prophylaxis during dental care and other adverse drug events. The critical information required were medical conditions or diagnosis, current medications, and allergies, which were gathered from patient reports. Furthermore, 88.2% (n=142) of respondents considered patient-reported histories reliable; however, they experienced challenges obtaining information from patients and physicians. Additionally, 70.2% (n=113) of respondents, especially those who currently access an HIE or electronic health record, were willing to use an HIE to access or share their patient’s information, and 91.3% (n=147) shared varying interests in such a service. However, usability, data accuracy, data safety, and cost are the driving factors in adopting an HIE.

**Conclusions:**

Patients’ medical histories are essential for dentists to optimize dental care, especially for those with chronic conditions. In addition, most dentists are interested in using an HIE to access patient medical histories. The findings from this study can provide an alternative option for improving communications between dental and medical professionals and help the health information technology system or tool developers identify critical requirements for more user-friendly designs.

## Introduction

More than 20 years ago, the first *US Surgeon General’s Report on Oral Health in America* established oral health as an essential component of overall health and well-being [1]. In 2021, the National Institutes of Health (NIH) reemphasized the importance of establishing integrated medical and dental care in their updated report on *Oral Health in America* [2]. In addition, the NIH identified integrating medical and dental records as critical to enhancing medical and dental care [2]. The integration of medical and dental records is gaining significance for several reasons. First, increased evidence during the last 3 decades indicates strong associations and shared risk factors between oral and systemic diseases such as diabetes and heart diseases [2,3]. Second, the siloed systems of dental and medical data create challenges in information sharing [2,4,5], often resulting in incomplete or inaccurate patient medical information, which may cause significant patient care and safety issues in dental care [6-9]. Third, recent studies have revealed discrepancies in medical conditions and medications in the electronic dental record (EDR) versus electronic health record (EHR) [3,6,10-14] and demonstrated substantial delays when dentists are required to request additional medical information from physicians [15]. Other reasons for the increased significance of integrated medical and dental records include the rapid development of information technologies, which provides a solid base for integration, and the impact of the COVID-19 pandemic, which proves the urgency and importance of integrating medical and dental records [16-18].

The use of an integrated EDR-EHR system has been growing in large health care organizations (HCOs) such as the Veteran Affairs health care systems, Department of Defense, health maintenance organizations, and federally qualified health centers, where medical and dental practices are colocated and share patient care and records [19-21]. Numerous studies have reported physicians using integrated EDR-EHR systems to refer patients to dentists and vice versa for preventive and comprehensive care [19,21-23]. However, for most dentists who work in small independent practices, patients continue to be the primary source of their medical history and dentists’ communications with medical providers are limited [24]. Nevertheless, the solo and small-group dental practices, which constitute 50% of the dental workforce [25], cannot adopt such integrated systems without being credentialed to a major HCO. It is also not practical for dental practices to have separate interfaces to different EHR systems, which may interfere with their clinical workflows and business processes, such as billings and regulatory policies [3].

With the support of several federal policies, such as the Health Information Technology for Economic and Clinical Health Act and the 21st Century Cures Act [26,27], and the financial incentives established by the Centers for Medicare and Medicaid Services [28,29], community and regional health information exchanges (HIEs) have expanded significantly since 2009. HIE systems provide another option for integrating medical and dental records [24]. In an earlier study by the team, we modeled 3 methods for dentists to access their patient’s medical histories: the patient-reported medical history followed by the optional medical consults method, the integrated EDR-EHR, and the HIE approaches [24]. Our models showed that the HIE approach could provide benefits for reducing unnecessary medical consults, avoiding the delay of care, improving information quality, and cutting additional technical and financial overheads for small independent practices. In addition, a report published in 2021 indicated a decrease in dentists working solo [25]. Nevertheless, an HIE-based integrated solution can help small and large group practices improve data completeness and compliance by obtaining data from multiple HCOs and taking advantage of expert services provided by an HIE. However, efforts to connect dentists with an HIE are minimal compared to the extensive studies on integrated EDR-EHR [19-22].

Despite the widespread interest in integrating dental and medical care, few studies have evaluated the opinions of practicing dentists on patient medical histories [26,30]. For example, a recent study published by the American Dental Association Clinical Evaluators Panel reported that most dentists gathered their patients’ medical history and medication list via patients’ self-report and recorded vital signs during dental visits [30]. However, questions remain on dentists’ information needs; their perception of the reliability of patient-reported medical history; satisfaction with the available information and the methods to gather this information; and their attitudes to other options, such as an HIE, to collect patient medical history.

Given this knowledge gap, we surveyed dentists in 1 US state about their information needs and practices concerning retrieving patient medical history information. Our objectives were to determine their information needs regarding patients’ medical information and their opinions on accessing it via an HIE.

## Methods

### Recruitment

We administered a web-based survey to the Indiana Dental Association (IDA) members from March 19, 2021, to April 30, 2021. All participants are general dentists or specialists either currently or previously practicing in the State of Indiana. We only included dentists in this survey because they are responsible for diagnosing and planning treatments, which also involve ruling out contraindications. The survey was administrated through the Indiana University–approved Qualtrics Experience Management platform XM. We sent emails to 2148 IDA members over 6 weeks, including 1 initial invitation, 5 reminders, and 1 final thank you note.

### Ethical Considerations

Participation in the survey was voluntary, responses were anonymous, and participants could only respond once (configurations blocked multiple responses in the web-based survey tool). Participants gave informed consent by accessing the link provided in the study invitation email. The patients were not compensated. This study received exemption approval from the Indiana University Institutional Review Board (Protocol #2012972646).

### Survey Construction and Validation

The survey included 27 questions covering 3 topics: 12 on demographics, 11 on information needs and gathering, and 4 on exchanging patient medical information (Multimedia Appendix 1). Demographic information included sex, years in practice, primary practice information (type of practice, general practitioner or specialist, typical procedures, patient age groups), and EDR use. The information needs and data gathering section included questions related to dentists’ information needs, existing methods for collecting information, and challenges in these approaches. Finally, the exchange of patient medical information section included dentists’ opinions on using an HIE-based information platform to receive and share patients’ medical information. The survey had 23 multiple-choice questions, two 0-10 Likert-scale questions, and 2 open-ended questions. We administered the survey after assessing the face validity and content validity of the questionnaire. The face validity was assessed with research team members who were not involved in the development of the survey questions, and the content validity was assessed with 3 dentists—2 from the Indiana University School of Dentistry and 1 from private practice. These tests ensured that the survey was appropriate, understandable, and could be completed within a reasonable time.

### Statistical Analysis

Data analysis included only completed responses. Partially answered surveys were eliminated from the final analysis. Data were summarized using frequencies and percentages for categorical variables and mean and SDs for continuous variables. Associations between characteristics of dentists (years in practice, dental professions, and current access to an HIE or a hospital or medical practice–based EHR [hereby referred to as HIE-EHR]) and their opinions on the importance and reliability of patients’ medical histories and perceptions of accessing patients’ medical histories via an HIE were examined using multivariable regression. The ordinal logistic regression model was used due to the ordinal nature of these variables. All statistical analyses were performed using SAS (version 9.4, The SAS Institute). *P*<.05 were considered statistically significant.

## Results

At the end of 6 weeks, 219 (10.2%) out of 2148 IDA members accessed the survey, and 188 (8.8%) members responded to at least 1 question, of which 161 (7.5%) members reached the end of the survey.

### Demographics

A total of 64.6% (102/158) of the respondents were male (Table 1). Their average years in practice was 25.72 (SD 13.52) years. A total of 74.5% (120/161) of the respondents were general practitioners, and the rest were dental specialists (Table 1). A total of 8 dental specialties were reported: oral and maxillofacial surgery (10/41, 24.2%), periodontics (8/41, 19%), orthodontics (7/41, 16.7%), pediatric dentistry (7/41, 16.7%), endodontics (4/41, 11.9%), operative dentistry (2/41, 4.8%), prosthodontics (2/41, 4.8%), and oral and maxillofacial pathology (1/41, 2.4%). The total percentage is more than 100% since some respondents reported more than 1 specialty.

**Table 1:** Characteristics of survey respondents.

Characteristics				Values
**Survey respondents**				
	**Sex (n=158), n (%)**			
		Female	56 (35.4)	
		Male	102 (64.6)	
	**Dental profession (n=161), n (%)**			
		General practitioner	120 (74.5)	
		Dental specialist	41 (25.5)	
	Years in practice (n=161), mean (SD)			25.72 (13.52)
**Survey respondents’ primary dental practices**				
	**Type (n=160), n (%)**			
		Private dental practice owner	100 (62.5)	
		Associate dentist of a private practice	26 (16.3)	
		Major dental care organizations such as dental schools and health maintenance organizations	19 (11.9)	
		Public health practice, community health center, or publicity-funded clinic (but not a federal facility)	2 (1.3)	
		Federal government facility (Veterans Affairs, Department of Defense, and Public Health Service)	2 (1.3)	
		Other	11 (6.9)	
	**Number of dentists (including the respondent; n=160), n (%)**			
		1	75 (46.9)	
		2-5	66 (41.3)	
		6-10	3 (1.9)	
		>10	16 (10)	
	**Number of hygienists (n=161), n (%)**			
		0	31 (19.3)	
		1-5	110 (68.3)	
		6-10	15 (9.3)	
		>10	5 (3.1)	
	**Use EDR^a^ to manage clinical data (n=161), n (%)**			
		Yes	128 (79.5)	
		No	33 (20.5)	
	**EDR system brands (n=126), n (%)**			
		Dentrix	45 (35.7)	
		EagleSoft	19 (15.1)	
		axiUm	14 (11.1)	
		OpenDental	10 (7.9)	
		SoftDent	10 (7.9)	
		Practice Works	6 (4.8)	
		Easy Dental	3 (2.4)	
		Other^b^	19 (15.1)	
	**Whether or not have access to a state-based health information exchange, exchange capability between dental software and electronic medical record system, or integrated dental-medical record system? (n=161), n (%)**			
		Yes	24 (14.9)	
		No	137 (85.1)	
	**Patient age distribution (%; n=157), mean (SD)**			
		18 years and younger	19.6 (23.3)	
		19-44 years	26.1 (13.8)	
		45-64 years	31.8 (15)	
		65 years and older	22.5 (13.1)	

^a^EDR: electronic dental record.

^b^Other EDR brands included Ascend (by Dentrix), Cloud9, Curve Dental, Denticon, DRM plus, DSN PerioExec, EPMS, MacPractice, Mconsent, Florida Probe, Mogo, NextGen, OMSvision, Ortho2 Edge, Practice Fusion, and Practice Web.

In all, 78.8% (126/160) of respondents reported working in private practices as owners or associate dentists (Table 1). Approximately half (75/160, 46.9%) of the respondents reported having 1 dentist in their primary dental practices, while 10% (16/160) of respondents reported their primary practice having more than 10 dentists. Most respondents’ (110/160, 68.3%) primary practices had 2-5 hygienists, while 19% (31/160) of respondents’ practices had no hygienists. The 3 most frequently performed procedures were diagnostic and preventive such as an examination, X-rays, scaling, prophylaxis, sealants, fluoride, etc (136/161, 84.4%); restorations or fillings (125/161, 78.1%); and tooth-supported or implant-supported crowns (105/161, 65.3%). The respondents served a diverse age group of the patient population, with an average of 19.6% (31/157) of patients 18 years or younger and 22.5% (35/157) of patients 65 years or older (Table 1).

About 4 in 5 (128/161, 79.5%) respondents reported using an EDR for not only billing or scheduling but also for clinical or patient data management. The top 3 brands of EDR were Dentrix (145/161, 35.7%), EagleSoft (19/161, 15.1%), and axiUm (14/161, 11.1%; Table 1).

### Dentists’ Opinions on the Importance of Medical Histories and Reliability of Patient-Reported Medical Histories

Almost all respondents (160/161, 99.5%) considered patients’ medical histories highly or moderately important during dental care (Table 2). They reviewed medical histories to (1) verify no contraindications exist to undergo a dental procedure (37/161, 23.2%), (2) confirm no need for antibiotic prophylaxis before the dental procedure (36/161, 22.7%), (3) rule out any allergies or adverse drug reactions (35/161, 22%), (4) assist with determining the prognosis of an oral disease or treatment outcomes (35/161, 21.7%), (5) detect normal and abnormal laboratory results (14/161, 8.4%), and (6) for other purposes (3/161, 1.9%). Only 1 respondent considered patients’ medical histories unimportant since they felt gathering medical history to be procedural and not essential for dental care. Regarding the reliability of patient-reported medical histories, 8% (n=13) of respondents considered them highly reliable, 79.5% (n=128) moderately reliable, and 12.4% (n=20) unreliable.

**Table 2:** Respondents’ opinions on the importance and reliability of patients’ medical histories and their perceptions of accessing patient history via an HIE^a^ (n=161).

Opinions				Values, n (%)
**Opinions to patients’ medical histories^b^**				
	**How important is obtaining patient’s up-to-date medical history for you?**			
		4	1 (0.6)	
		7	3 (1.9)	
		8	13 (8.1)	
		9	18 (11.2)	
		10: Extremely important	126 (78.3)	
	**How reliable is the patient-reported medical history?**			
		2	2 (1.2)	
		3	3 (1.9)	
		4	4 (2.5)	
		5	11 (6.8)	
		6	29 (18.0)	
		7	57 (35.4)	
		8	42 (26.1)	
		9	11 (6.8)	
		10: Extremely reliable	2 (1.2)	
**Perceptions of accessing patient history via an HIE**				
	**Do you think access to such a system would be useful?**			
		1: No	8 (5.0)	
		2: Maybe	40 (24.8)	
		3: Yes	113 (70.2)	
	**Would you consider using it to access your patient’s medical information?**			
		1: No	9 (5.6)	
		2: Maybe	40 (24.8)	
		3: Yes	112 (69.6)	
	**Would you allow other health care providers to access clinical information about your own patients?**			
		1: No	15 (9.3)	
		2: Maybe	47 (29.2)	
		3: Yes	99 (61.5)	
	**What is your interest to participate in a service to access such as a system?**			
		1: Not interested at all	15 (9.3)	
		2: Slightly interested	19 (11.8)	
		3: Moderately interested	58 (36.0)	
		4: Very interested	39 (24.2)	
		5: Extremely interested	30 (18.6)	

^a^HIE: health information exchange.

^b^10-level Likert scale was used with ranges as follows: 1-5=not important or reliable, 6-8=moderately important or reliable, and 9-10=highly important or reliable.

### Dentists’ Information Needs Regarding Their Patient’s Medical History

The 3 most needed information categories for new and existing patients were medical conditions or diagnosis, current medications, and allergies. Other categories included hospitalizations in the last 2 years, substance abuse, procedures in the previous 5 years, laboratory results from the last 6 months, and immunization records. The respondents evaluated the information needs of new and existing patients separately, and there were no significant differences in the results (Table 3).

**Table 3:** Dentists’ most needed patient medical information during dental care.

Patient medical information	Existing patients (n=161), n (%)	New patients (n=161), n (%)
Medical condition or diagnoses	150 (93.2)	153 (95)
Current medications	141 (87.6)	148 (91.9)
Allergies	138 (85.7)	143 (88.8)
Substance abuse	76 (47.2)	81 (50.3)
Hospitalization in the last 2 years	70 (43.5)	68 (42.2)
Procedures in the last 5 years	44 (27.3)	49 (30.4)
Laboratory results from the last 6 months	17 (10.6)	17 (10.6)
Immunization records	4 (2.5)	3 (1.9)
Others	3 (1.9)	5 (3.1)

### Dentists’ Access to Their Patient’s Medical History

We also asked the dentists how they collected patient-reported medical histories and obtained additional information if needed. Paper-based health history forms constituted the most used method (127/161, 78.9%), followed by web-based health history forms (62/161, 38.5%) and electronic devices such as tablets (35/161, 21.7%). The total percentage is more than 100% since some respondents reported using more than 1 method. The top 3 challenges in collecting patient-reported medical history were as follows: (1) patients do not remember or recall medication names and dosage (156/161, 96.9%); (2) patients do not recall previous procedures and medical conditions (129/161, 80.1%); and (3) patients’ reluctance to share their medical history (84/161, 52.2%). When the respondents needed additional information, most (158/161, 98%) contacted physicians’ offices or health care providers directly via phone, fax, or email. Other communication methods included paper-based medical consult forms through the patient (46/161, 28.6%), patient’s pharmacy (39/161, 24.2%), state-based HIE (19/161, 11.8%), exchange capability between dental software and electronic medical record system (5/161, 3.1%), integrated dental-medical record system (4/161, 2.5%), and other (9/161, 5.6%). However, they experienced challenges such as the need for multiple attempts (97/161, 60.2%), not receiving information on time (80/161, 49.7%), physician offices being nonresponsive (66/161, 41.0%), need to contact numerous providers or specialists (55/161, 34.2%), need for patient intervention (44/161, 27.3%), and not receiving requested information (35/161, 21.7%).

### Dentists’ Perceptions of Accessing Patient History via an HIE

A total of 69.6% (113/161) of respondents considered access to a regional HIE useful (Table 2). If such a system were available, 69.9% (n=113) of the respondents would consider using it to access their patient’s medical information, and 61.5% (n=99) would be willing to allow other health care providers to access their patients’ clinical information (Table 2). Furthermore, 91.3% (n=147) of the respondents expressed various interests in participating in a service to access an HIE (Table 2). However, they expressed concerns over the design and implementation of such a system, including data accuracy, data security and HIPAA (Health Insurance Portability and Accountability Act) compliance, cost of implementation (both time and money), and system usability.

The association between respondent characteristics (including dental profession, number of years in practice, and current access to an EHR or HIE) and their opinions on the importance and reliability of patients’ medical histories and perceptions of accessing patients’ medical histories via HIE based on multivariable ordinal logistic regression is displayed in Table 4. Dental profession type (general practitioner vs dental specialist) does not significantly affect one’s opinions toward the importance (*P*=.98) and reliability (*P*=.31) of patients’ medical history. However, respondents with more than 40 years in practice were less likely to consider obtaining up-to-date patient information important compared to those with less than 40 years in practice (odds ratio [OR] 0.351, 95% CI 0.139-0.889; *P*=.03) and more likely to think self-reported information to be reliable (OR 2.267, 95% CI 1.011-5.084; *P*=.047). In addition, respondents with access to an HIE-EHR were more likely to consider obtaining up-to-date patient information important compared to those who do not have access to an HIE-EHR (OR 2.590, 95% CI 1.080-6.209; *P*=.03). Regarding the respondents’ perceptions of using an HIE to access patients’ medical histories, we found that dental specialists were more interested than general practitioners in participating in service to access patient information via an HIE (OR 2.267, 95% CI 1.174-4.378, *P*=.02). Compared to respondents without current access to an HIE-EHR, those with access to an HIE-EHR were more likely to think it worthwhile to access such a system (OR 6.306, 95% CI 2.671-14.886; *P*<.001), more likely to consider using such a system to access their patient’s information (OR 5.538, 95% CI 2.379-12.892; *P*<.001), more likely to allow other providers to access their patient’s data (OR 2.943, 95% CI 1.342-6.456; *P*=.007), and more interested in participating in service to access such a system (OR 3.894, 95% CI 1.844-8.222; *P*<.001).

**Table 4:** Impact of respondents’ demographics on their opinions on patient medical history and perceptions on accessing patient medical information via an HIE^a^.

Patient medical information	Dental specialist vs general practitioner		>40 vs ≤40 years in practice			Have access to HIE-EHR^b^ vs no access	
	OR^c^ (95% CI)	*P* value	OR (95% CI)	*P* value	OR (95% CI)		*P* value
How important is obtaining a patient’s up-to-date medical history for you?	0.988 (0.401-2.437)	.98	0.351 (0.139-0.889)	.03^d^	2.590 (1.080-6.209)		.03^d^
How reliable is the patient-reported medical history?	0.713 (0.374-1.360)	.31	2.267 (1.011-5.084)	.047^d^	1.135 (0.554-2.327)		.73
Do you think access to such a system would be useful?	1.567 (0.674-3.643)	.30	2.435 (0.821-7.217)	.11	6.306 (2.671-14.886)		<.001^d^
Would you consider using it to access your patient’s medical information?	2.187 (0.908-5.264)	.08	1.577 (0.577-4.309)	.37	5.538 (2.379-12.892)		<.001^d^
Would you allow other providers to access clinical information about your own patients?	1.311 (0.623-2.759)	.48	1.517 (0.602-3.825)	.38	2.943 (1.342-6.456)		.007^d^
What is your interest in participating in a service to access such a system?	2.267 (1.174-4.378)	.02^d^	1.609 (0.722-3.585)	0.24	3.894 (1.844-8.222)		<.001^d^

^a^HIE: health information exchange.

^b^EHR: electronic health record.

^c^OR: odds ratio.

^d^*P*<.05 were considered statistically significant.

## Discussion

### Principal Findings

We surveyed Indiana dentists to determine their information needs regarding patients’ medical histories and their opinions of accessing patient-specific medical information via a community or regional HIE. The survey respondents’ demographics distribution closely matched the dentists’ demographics in the 2020 Indiana oral health workforce data report [31]. In addition, the response rate of 7.5% (161/2148) is comparable to previous surveys of health care professionals, especially web-based surveys [32-34]. The results demonstrated dentists’ high priority in obtaining their patients’ medical diagnoses or conditions, medication histories, and allergies to provide optimum dental care. The survey respondents also reported challenges in getting medical information from patients and medical providers, although they considered patient-reported medical histories moderately or highly reliable. It is also significant that 70% (112/160) of surveyed dentists who work primarily in community practices (Table 1) expressed willingness to use and participate in a service to access and share their patients’ medical histories via an HIE.

Nevertheless, the participants commented that usability, data accuracy, data safety, and implementation costs would drive dental providers’ use of such services. Integration of dental and medical record data is critical to promote communication and care coordination between dental and medical providers and has gained tremendous attention in recent years [2]. However, existing studies only highlight case studies of integrating dental and medical care in large health care systems [19-21]. Through this study, we determined community practice dentists’ information needs and attitudes toward accessing patient medical information via an HIE. These study results contribute to dental professionals’ high-priority information needs and HIE functionalities for successfully using the expanding HIE network in the United States and other countries. In the sections below, we discuss the relevant findings in detail.

Dentists with <40 years of experience or having access to an HIE-EHR system felt patients’ medical histories were more critical than those with >40 years of experience, even though almost 90% (145/161) of the dentists considered patients’ medical histories essential (Table 2). This difference could be because, until 2 decades ago, only limited information technology existed for dentists to access their patient’s medical information except for patient-reported medical history and medical consults. This limited access to EHR data may explain why dentists with more than 40 years in practice were more likely to think patient-reported information as reliable (Table 4). Additionally, dentist respondents who already have access to an HIE-EHR system may benefit more from their patients’ medical histories since they have easier access to the information and may have better quality of information.

Our survey found that the most needed information categories were medical conditions or diagnosis, current medications, and allergies (Table 3), which was consistent with a previous survey [26]. Together these findings showed that some categories of patient medical information were more helpful to dentists during dental care. These findings can also be used to optimize the user interface design in either an EDR-EHR system or an HIE to avoid information overload. However, our team’s earlier studies on medical consults discovered that dentists’ most requested information categories were laboratory values and written diagnostic reports, followed by recommendations or medical clearances [15]. The inconsistency of these results indicates that dentists’ information needs can evolve based on access to relevant information. As they gain access to EHR information, they can ask more specific and informed questions when consulting their medical colleagues, leading to increased responses from medical colleagues. This improved information access may enhance dentists’ patient management and treatment planning. The results also indicated dentists’ information needs for new and existing patients were almost identical (Table 3). Future studies should continue investigating dentists’ information needs as they gain direct access to patients’ up-to-date medical information via an EDR-EHR system or an HIE.

The survey respondents, especially those with access to an HIE-EHR, showed clear interest in using the HIE to optimize the information collection process (Tables 2 and 3). For instance, 11.8% (19/161) of the respondents reported access to a state-based HIE, which was higher than expected. This higher access rate could be attributed to dental providers’ access to state-wide information systems, such as Indiana’s Prescription Drug Monitoring Program, and may have mistaken it for an HIE. Nonetheless, several state-wide HIEs are promoting dentists’ use of HIEs to improve access to patient information [35-37]. However, the overall use during dental care remains low. For instance, a study of New York dentists’ use of the Rochester regional HIE demonstrated a 0.17% rate of use of the HIE during dental encounters [35]. This low use is not surprising given that the use of community HIEs, even by nondental providers, is still growing, with 1% to 5% use in all patient visits. In the New York dentists’ study, they accessed the HIE primarily for patients with chronic conditions, gingival and periodontal diagnosed diseases, and during the first dental visit [35]. The most frequently visited sections were the laboratory and radiology sections within the HIE, which is consistent with our earlier study results of dentists’ medical consult requests [15].

Although the emergence of community and vendor-supported HIEs has improved medical providers’ timely access to patient information [38-41], inefficient and cumbersome processes and poor user experiences are significant barriers to HIE use [42,43]. Previous studies in medical settings reported that some HIEs require users to have multiple logins; interrupt their workflow; and display overloaded and poorly arranged information [42,43]. Our study respondents expressed similar concerns about the usability of HIEs, such as difficulty accessing data, information overload, and nonintuitive interface designs that could prevent dentists’ use of HIEs. Therefore, future HIE tools’ design and development should focus on the accuracy and integration of the data (content) and the information display and navigation (presentation). Few respondents including those willing to use an HIE expressed concerns about accessing patients’ medical histories via an HIE due to data safety and HIPAA compliance concerns. This issue needs to be addressed both at the technical level with more new tools and methods to ensure safe data sharing and exchanging and at the regulatory level with new protocols and rules to support the use of HIEs. Furthermore, most respondents agreed that patients should be able to control the use of their health care information, and their consent must be received before any information exchange and sharing occur.

### Limitations

This study only invited Indiana dentists who are IDA members. A more geographically diversified pool of participants may help improve the results’ validity and generalizability. In future studies, we also want to include other dental professionals, such as dental hygienists and dental assistants. We are aware of the relatively low response rate to the survey, which is not rare in surveys of health care professionals, especially web-based surveys [32-34]. This was an exploratory study and our initial step to determine dentists’ information needs and to help improve their information access. Based on the results of this survey, we will conduct key informant interviews and focus group studies to include a broader group of participants. Another limitation was that dentists may not be familiar with some of the terminologies used in the survey such as state-based HIEs. Terminology definitions and examples should be included in future survey designs.

### Conclusions

Patients’ medical histories are essential for dentists to provide high-quality dental care. In addition, information such as medical conditions or diagnosis, current medications, and allergies are more relevant to dentists’ clinical decision-making. Paper-based health history forms and medical consults are still the most widely used methods to gather information. However, electronic forms and integrated systems are gaining attention to have direct access to information. Most dentists are interested in using an HIE to access patient medical histories. The findings from this study can provide an alternative option for improving communications between dental and medical professionals and help the health information technology system or tool developers identify critical requirements for more user-friendly designs.

## Appendix

Multimedia Appendix 1 Survey on how essential patient medical history is for dental care.

## Abbreviations

EDR: electronic dental record
EHR: electronic health record
HCO: health care organization
HIE: health information exchange
HIPAA: Health Insurance Portability and Accountability Act
IDA: Indiana Dental Association
NIH: National Institutes of Health
OR: odds ratio

## Data Availability

The data sets generated and analyzed during this study are available from the corresponding author on reasonable request. Requests may need approval from Indiana University.
