# Supplementary material for: Dentists’ Information Needs and Opinions on Accessing Patient Information via Health Information Exchange: Survey Study
Source: JMIR Form Res. 2024 Jan 11;8:e51200. doi: 10.2196/51200 (PMC10811575; doi:10.2196/51200)
Supplement: Multimedia Appendix 1 [file formative_v8i1e51200_app1.pdf]

Appendix A

How essential is patient medical history for dental care?

Thank you for participating in this survey. Your responses will help us understand the importance of medical history to dental practitioners, how they are collected, barriers and facilitators. The survey should take approximately 10 minutes. The survey is divided into 3 sections: 1) practice demographics, 2) information gathering and 3) information exchange between practitioners.

1. Demographics

1.1 Gender?

- ○ Female
- ○ Male
- ○ Prefer not to say
- ○ Other... (please specify) ________________________________

1.2 Year of graduation?

____

1.3 How long have you been in practice? (Please input a numerical value)

____

1.4 Are you a general practitioner or a specialist?

- ○ General practitioner
- ○ Dental specialist

1.5 If you are a dental specialist, please select the appropriate specialty. (Please select all that apply.)

- ☐ Dental Public Health
- ☐ Endodontics
- ☐ Operative Dentistry
- ☐ Oral and Maxillofacial Pathology
- ☐ Oral and Maxillofacial Radiology
- ☐ Oral and Maxillofacial Surgeon
- ☐ Oral Medicine
- ☐ Orthodontics
- ☐ Pediatric Dentistry
- ☐ Periodontics
- ☐ Prosthodontics
- ☐ Other... (please specify) ____

1.6 Which category below best describes your primary dental practice?

- ○ Owner of a private practice
- ○ Associate or employee of a private practice
- ○ Managed care organization
- ○ Public health practice, community health center, or publicity-funded clinic (but not a federal facility)
- ○ Federal government facility (VA, Department of Defense, Public Health Service)
- ○ Dental school, academic dental institution, facility staffed by the dental school
- ○ Other... (please specify) ____

1.7 How many dentists (including you) work at your practice?

- ○ 1
- ○ 2 to 5
- ○ 6 to 10
- ○ More than 10

1.8 How many hygienists does your practice employ?

- ○ 0
- ○ 1 to 5
- ○ 6 to 10
- ○ More than 10

1.9 In a typical week, what are the most common procedures that you devote most of your time? (Please select at least three)

- ☐ Diagnostic and preventive (exam, x-rays, scaling, prophylaxis, sealants, fluoride, etc.)
- ☐ Restorative (fillings)
- ☐ Crowns (tooth-supported and implant-supported)
- ☐ Extractions
- ☐ Implant surgery
- ☐ Periodontal therapy (surgical)
- ☐ Endodontic therapy
- ☐ Orthodontics (including Invisalign)
- ☐ Cosmetic Dentistry
- ☐ Full-mouth reconstruction
- ☐ Maxillofacial prosthodontics
- ☐ TMD and sleep apnea appliance
- ☐ Other... (please specify) ____

1.10 Please indicate the approximate percentage of patients in your practice who are: (Total should be 100%)
Children & Teenagers (1 to 18 years): ________
Young Adults (19 to 44 years): ________
Middle Aged Adults (45 to 64 years): ________
Older Adults (65 or older): ________
Total: _________

1.11 Do you use electronic dental records to manage clinical/patient data (not just billing/scheduling)?

- ○ Yes
- ○ No

1.12 What brand of electronic dental record systems do you use?

- ◯ axiUm
- ◯ Dentrix
- ◯ EagleSoft
- ◯ Easy Dental
- ◯ Epic Wisdom
- ◯ Open Dental
- ◯ Practice Works
- ◯ SoftDent
- ◯ Other... (please specify) ____

2. Information Gathering

2.1 When a patient makes a dental appointment, which types of information do you **Always Request** and which types do you ask **As Needed**?

	Always Request (1)	As Needed (2)
Insurance Information	○	○
Chief Complaint	○	○
Medical History	○	○
Medication History	○	○
Allergies	○	○
Immunization Records	○	○
Past/Present Dental History	○	○
Radiographs/Images	○	○
Other... (please specify)	○	○

2.2 How important is obtaining patient's up-to-date medical history for you?

- ○ 0
- ○ 1
- ○ 2
- ○ 3
- ○ 4
- ○ 5
- ○ 6
- ○ 7
- ○ 8
- ○ 9
- ○ 10

2.3 Why is obtaining your patient's up-to-date medical history important? (Please select all that apply.)

- ☐ Assists with determining prognosis of an oral disease or treatment outcome
- ☐ Confirms no contraindications to undergo a dental procedure
- ☐ To confirm the need for antibiotic prophylaxis before dental procedures
- ☐ Rule out any allergies or adverse drug reactions

- [ ] Detecting normal and abnormal laboratory results
- [ ] Other... (please specify) ____

2.4 Why is obtaining your patient's up-to-date medical history **NOT** important? (Please select all that apply.)

- [ ] Patients in my practice do not have underlying medical condition/s
- [ ] Gathering medical history is procedural and is not essential for dental care
- [ ] Other... (please specify) ____

2.5 How do patients report their medical history at your practice? (Please select all that apply.)

- [ ] Paper-based health history forms
- [ ] Online health history forms
- [ ] Electronic devices (such as tablets)
- [ ] Other... (please specify) ____

2.6 How often do you update your patient's medical history form?

- ○ Every patient visit
- ○ Once every 6 months
- ○ Once a year
- ○ Once in two years
- ○ Once in five years
- ○ Other... (please specify) ____

2.7 How reliable is the patient-reported medical history?

- ○ 0
- ○ 1
- ○ 2
- ○ 3
- ○ 4
- ○ 5
- ○ 6
- ○ 7
- ○ 8
- ○ 9
- ○ 10

2.8 What challenges do you experience when collecting medical history from patients? (Please select all that apply.)

- ☐ Patients do not remember/recall medication names and dosage
- ☐ Patients do not recall previous procedures and/or medical conditions
- ☐ Patients reluctant to share their medical history
- ☐ Patients do not have recent lab results
- ☐ Patients cannot communicate or write in English
- ☐ Patients are mentally or physically challenged to provide up-to-date medical history
- ☐ Other... (please specify) ______________________________

2.9 (a) What patient-specific medical information do you wish to have access when providing dental care for **a new patient** in your practice? (From **Most Wanted** to **Least Wanted**)

	**New Patient**		
	Most Wanted (1)	Wanted (2)	Least Wanted (3)
Medical conditions/diagnoses	○	○	○
Allergies	○	○	○
Immunization Records	○	○	○
Medications: current	○	○	○
Procedures in the last five years	○	○	○
Hospitalizations in the last two years	○	○	○
Substance abuse	○	○	○
Laboratory results from last six months	○	○	○
Other... (please specify)	○	○	○

2.9 (b) What patient-specific medical information do you wish to have access when providing dental care for **an existing patient** in your practice? (From **Most Wanted** to **Least Wanted**)

	**Existing Patient**		
	Most Wanted (1)	Wanted (2)	Least Wanted (3)
Medical conditions/diagnoses	○	○	○
Allergies	○	○	○
Immunization Records	○	○	○
Medications: current	○	○	○
Procedures in the last five years	○	○	○
Hospitalizations in the last two years	○	○	○
Substance abuse	○	○	○
Laboratory results from last six months	○	○	○
Other... (please specify)	○	○	○

2.10 If medical history is **NOT** patient-reported or you need additional information, then how do you obtain this information? (Please select all that apply.)

- ☐ Contact physician's office or healthcare provider by phone
- ☐ Contact physician's office or healthcare provider by fax
- ☐ Contact physician's office or healthcare provider by secure email
- ☐ Paper-based medical consult form through the patient
- ☐ Patient’s pharmacy
- ☐ State-based health information exchange
- ☐ Exchange capability between dental software and electronic medical record system
- ☐ Integrated dental-medical record system
- ☐ Commonwell Health Alliance or another national exchange
- ☐ Other... (please specify)____

2.11 What challenges have you experienced when contacting healthcare providers or healthcare facilities to obtain a patient's medical history? (Please select all that apply.)

- ☐ I don't have any problems receiving patients' medical history from healthcare providers/facilities
- ☐ Do not receive information on time
- ☐ Physician offices are non-responsive
- ☐ Require multiple attempts
- ☐ Need patient intervention
- ☐ Do not provide requested information
- ☐ Have to contact multiple providers or specialists
- ☐ Other... (please specify) ____

3. Information Exchange between Practitioners

The increasing use of electronic health record systems is enabling health care providers to share information through community/regional healthcare information exchange, like the Indiana Health Information Exchange (IHIE). For instance, you could access the medical and medication history of a new patient directly from his or her medical record system through such an exchange.

3.1 Do you think access to such a system would be useful, provided it is secure and confidential? (You may add your reasons/concerns in the text box below each option.)

- ○ Yes____
- ○ Maybe____
- ○ No____

3.2 If such a system was available, would you consider using it to access your patient's medical information? (You may add your reasons/concerns in the text box below each option.)

- ○ Yes ____
- ○ Maybe ____
- ○ No ____

3.3 Would you allow other health care providers to access clinical information about your own patients? (You may add your reasons/concerns in the text box below each option.)

- ○ Yes____
- ○ Maybe ____
- ○ No____

3.4 What is your interest to participate in a service to access such as a system?

- ◯ Extremely interested
- ◯ Very interested
- ◯ Moderately interested
- ◯ Slightly interested
- ◯ Not interested at all
